# Changes in physicochemical profiles and quality of apple juice treated by ultrafiltration and during its storage

**DOI:** 10.1002/fsn3.1593

**Published:** 2020-04-28

**Authors:** Ming Cai, Chunfang Xie, Yuqing Lv, Kai Yang, Peilong Sun

**Affiliations:** ^1^ Department of Food Science and Technology Zhejiang University of Technology Hangzhou China

**Keywords:** apple juice, polyphenol, quality, storage, ultrafiltration

## Abstract

Effects of various factors, such as membrane materials, molecular weight cutoff, transmembrane pressure (TMP), and cross flow rate (CFR) on flux and physicochemical properties of apple juice during ultrafiltration and storage have been investigated. Clarity, color, total phenols, total proteins, total sugars, total soluble solids (TSS), pH, and some specific polyphenols of juices were evaluated. Results show that at conditions of PES‐10 kDa, CFR 30 L/hr, and TMP 0.75 MPa, a clarified juice obtained with color 0.15 A, clarity 96.94%*T*, TSS 9.55 °Brix, pH 4.2, and total phenols, total proteins, and total sugars were 64.12 and 13.20 μg/ml and 50.70 mg/ml, respectively. Chlorogenic acid, epicatechin, phloridzin, catechin, and caffeic acid decreased differently from 32.63, 17.33, 3.25, 7.58, and 0.75 μg/ml to 17.24, 12.38, 1.79, 5.27, and 0.25 μg/ml, respectively. Storage in refrigeration for 4 weeks, clarity, total sugars, and total phenols reduced by 2.5%, 6.4%, and 16.6%, respectively, while TSS increased by 3.1%.

## Graphical abstract

Clarity, color, total phenols, total proteins, total sugars, total soluble solids, pH, and some specific polyphenols of juices changed in ultrafiltration. Chlorogenic acid, epicatechin, phloridzin, catechin, and caffeic acid decreased differently after ultrafiltration. Storage of ultrafiltrated juice in refrigeration for 4 weeks, clarity, total sugars, and total phenols reduced by 2.5%, 6.4%, and 16.6%, respectively, while TSS increased by 3.1%.

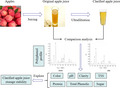

## 1 INTRODUCTION

As a health drink, apple juice stored nutrients, minerals, and micronutrients in apples and can be quickly absorbed by human body (Gerhauser, 2008). Clarified apple juice is popular among consumers because of its unique light transmittance, flavor, and taste. Some typical technologies, such as clarification agents, enzymatic methods, and membrane techniques have been widely used for clarification of apple juice.

Fining agents, such as gelatin, bentonite, silicasol, and diatomaceous earth, could create some problems of environmental impact due to their disposal. Addition of these clarifiers might affected some active ingredients loss and change the characteristics of juices (Vaillant et al., 1999). Enzyme treatment refers to the enzymatic hydrolysis of some components of juices with enzyme preparation. It can not only improve the yield and taste of juice, but also reduce the viscosity and color. However, enzyme treatment was time consuming and the optimal treatment conditions were difficult to be controlled (Girard & Fukumoto, 2000).

In 1977, Heatherbell, Short, and Strubi (1977) successfully applied ultrafiltration (UF) technology to produce a stable clarified juice. Accordingly, membrane technology as a non‐thermal technique has been emerged as a substitute to traditional juice clarification techniques because of low temperature, less operating cost, and less manpower. Additionally, it involves no phase change or chemical agents. UF is the most widely used membrane technology for clarification of fruit and vegetable juice in juice industry. Some studies found the application of UF to apple and lemon juices were successful, with reductions in color (99%) and viscosity (98%), subsequently achieving a high level of clarity (De‐Bruijn et al., 2003; Maktouf et al., 2014; Mirsaeedghazi, Emam‐Djomeh, Mousavi, Aroujalian, & Navidbakhsh, 2009; Toker, Karhan, Tetik, Turhan, & Oziyci, 2013; Warczok, Ferrando, Lopez, & Guell, 2004). Additionally, UF could be used to concentrate of phenolic compounds in juice, successfully in retaining a high percentage (85%) of polyphenols in its retentate (Conidi, Cassano, Caiazzo, & Drioli, 2017). But in most of these investigations, the changes of main ingredients during the processes have not been demonstrated clearly. Accordingly, the main components in juices, especially polyphenols can be affected by the membrane treatment. It is necessary to understand the changes of physicochemical profiles of juices by UF treatment, especially the phenolics. And the stability of ultrafiltrated juice during storage should be also demonstrated.

In this study, effects of various factors on apple juice during UF have been investigated. Changes of physicochemical properties and some specific phenolic compounds during the process and its storage have been demonstrated.

## 2 MATERIALS AND METHODS

### 2.1 Materials and reagents

Fresh “Fuji” apples were purchased from a local market (Zhejiang, China). The apples were washed, peeled, and the cores were also removed, after which the apple flesh was cut into slices. Immediately, the slices were immersed into 0.6% ascorbic acid solution to avoid the enzymatic browning. Afterward, the slices were squeezed by a juice extractor (JYL‐C022E, Joyoung). The juice was collected and filtered with a 100 mesh filter. After sterilization at 98°C for 30 s and filled in brown glass bottles, the juice was cooled to room temperature (25°C) for further UF immediately.

Folin–Ciocalteu reagent, sulfuric acid, phenol, ammonium acetate, bovine serum albumin, and ethyl acetate, all of analytical grade, were purchased from Aladdin. Chlorogenic acid (>97%), caffeic acid (>97%), catechin (>97%), epicatechin (>97%), and phloridzin (>97%) were purchased from Shanghai Yuanye Bio‐Technology Co. Ltd. Coomassie brilliant blue was purchased from Shanghai Baoman Co. Ltd.

### 2.2 UF membranes and system

**FIGURE 1:**
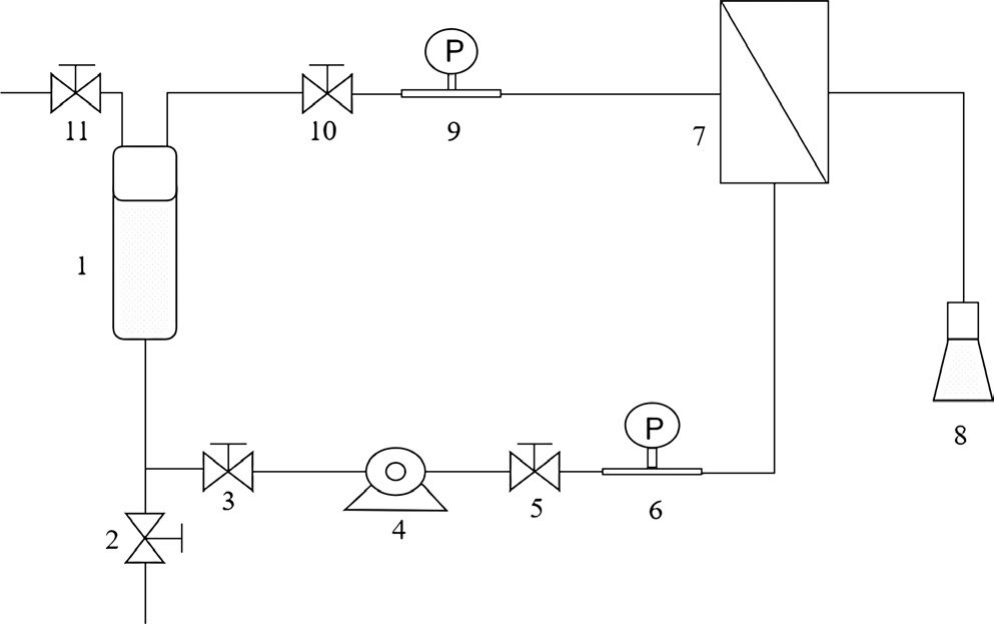
Schematic diagram of UF system. (1) feed tank; (2, 3, 5, 10, 11) retentive valve; (4) pump; (6,9) pressure gauge; (7) membrane module; (8) filtrate vessel

Five membranes with different materials and molecular weight cutoff (MWCO), as shown in Table 1, were employed in this study. The selection of MWCO was referred to the literatures (He, Ji, & Li, 2007; Onsekizoglu, Bahceci, & Acar, 2010) and our preliminary experiments. The schematic diagram of UF system is shown in Figure 1.

**TABLE 1:** Properties of UF membranes

Membrane	Material	MWCO (kDa)	Brand
M1	PAN	50	SEPRO
M2	PVDF	50	KOCH
M3	PES	50	NADIR
M4	PES	10	KOCH
M5	PES	5	KOCH

Abbreviations: PAN, polyacrylonitrile; PES, polyethersulfone; PVDF, polyvinylidene fluoride.

### 2.3 UF experiments

Two liter original juice was ultrafiltered for 30 min at 25°C. Cross flow rate (CFR) of 15, 30, and 45 L/hr, and transmembrane pressures (TMP) of 0.25, 0.5, and 0.75 MPa were employed. Permeate volume during process was recorded, and flux was calculated according to the following equation (Mello, Petrus, & Hubinger, 2010; Toh, Lim, & Livingston, 2007).

(1) $$J_{v}=\frac{\Delta V}{A_{m}t}$$

where $J_{v}$ is the permeate flux during UF process (L/(m^2^·hr)), Δ*V* is the permeate volume (L) collected at the same interval *t* (hr) and *A*_m_ is the active area of membrane (*A*_m_ = 2.38 × 10^–3^ m^2^).

Effects of different membrane materials, MWCO, flow rates, and TMPs on the quality of juice were investigated.

### 2.4 Physicochemical analysis

Color of fruit juice was measured by a spectrophotometer at 420 nm according to a published method (Rai et al., 2006).

According to percentage of transmittance (%*T*), clarity was measured by the method with some modification according to the following equation.

(2) $$\%T=100\times 10^{-A}$$

where *A* is the optical absorbance at a wavelength of 660 nm.

Total soluble solid (°Brix) was measured using Abbe refractometer as described by Ranganna (2005).

pH value of juice was measured by a multi‐parameter pocket tester (Allometrics, Inc.).

### 2.5 Determination of total phenolic

Total phenolic compounds in apple juice were determined by the Folin–Ciocalteu colorimetric method (Vasco, Ruales, & Eldin, 2008) with some modifications. 0.2 ml sample aliquot was mixed with 1 ml of a 10 fold diluted Folin–Ciocalteu reagent and 0.8 ml 7.5% sodium carbonate. The mixture was allowed to stand for 30 min at room temperature, measured at 760 nm by a UV‐visible spectrophotometer (V‐1800PC). Gallic acid solutions with concentrations ranging from 10 to 100 mg/L were used for calibration, and results were expressed as mg/L gallic acid equivalent (GAE).

### 2.6 Determination of total proteins

Total proteins were determined according to Bradford method (Popescu, MăRghitaş, & Bobiş, 2009). Principle of the Bradford Protein Assay is based on an absorbance maximum at 595 nm for Coomassie brilliant blue G‐250 (CBBG) when binding to protein occurs. The bovine serum albumin (BSA) as standard protein (10 mg) was dissolved in 10 ml 0.2 M phosphate buffered saline (PBS, pH 7.4) to be a concentration of 1 mg/ml as stock. 1, 2, 3, 4, and 5 μg/ml protein standards were prepared from the stock solution for the standard assay. One hundred milligram CBBG was dissolved in 50 ml 95% ethanol. One hundred milliliter phosphoric acid (85% w/v) was added, and the solution was diluted to be 1 L with deionized water and filtered twice immediately. One milliliter protein standards were mixed with 5 ml CBBG dye. After being incubated for 5 min, the absorbance at 595 nm was measured.

### 2.7 Determination of total sugars

Total sugars were analyzed by phenol‐sulfuric acid method (Masuko et al., 2005) with some modifications. 1.0 ml samples diluted with 1.0 ml distilled water were placed in a tube. One milliliter phenol solution was added, shaken, following added 5 ml concentrated sulfuric acid, rapidly. The mixture heated for 5 min at 90°C in a static water bath. After cooling to a room temperature for 20 min, the absorbance was measured at 490 nm.

### 2.8 HPLC analysis of polyphenols compositions

HPLC (Waters 1525) was used to identify and quantify the individual phenolics as described by Mello et al. (2010) with some modifications. It was performed using a reversed‐phase Agilent Zorbax Eclipse Plus‐C18 column (250 × 4.60 mm, 5 μm, Waters) according to the following conditions: flow rate = 1 ml/min; *T* = 30°C; *λ* = 280 nm. Mobile phase was methanol as solvent A and 1.0% acetic acid as solvent B, carried out by the following linear gradient: 0–10 min, 5%–20%A; 10–30 min, 20%–35%A; 30–40 min, 35%–45%A; 40–45 min, 45%–60%A; 45–50 min, 60%–5%A. Chlorogenic acid, caffeic acid, catechin, epicatechin, and phloridzin were used as comparison standard phenolic substances to determine various phenolic in apple juice by matching the retention time and their spectral characteristics and quantified by peak area.

### 2.9 Storage study

Ultrafilted apple juice was transferred into a sterile brown glass bottle with a sterile measuring cylinder and stored in a refrigerator at 4°C for 4 weeks in the dark. Changes of physicochemical properties, total proteins, polyphenols total sugars were determined weekly.

### 2.10 Statistical analysis

Each experiment was conducted in triplicate. The data were processed and analyzed by using OriginPro 8, and the data were expressed by mean standard deviation.

## 3 RESULTS AND DISCUSSION

### 3.1 Effects of various conditions on permeate flux

**FIGURE 2:**
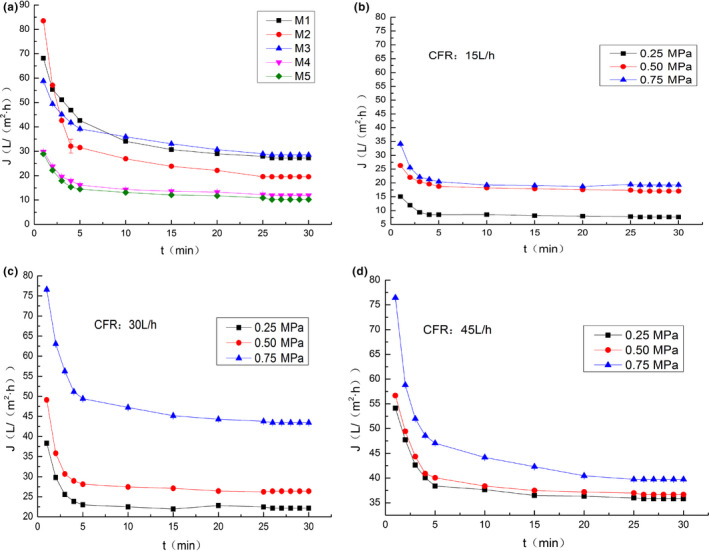
Effect of operating conditions on permeate flux: (a) different membranes; (b), (c), (d) different transmembrane pressure, and cross flow rate

Figure 2a shows that the fluxes of clarified juice decreased sharply at the initial 5 min, and they became stable after 30 min. The sharp drop of flux at initial stage was mainly caused by adsorption and membrane pore obstruction of compounds in juice, and in the latter stage was due to the accumulation of foulants on membranes surface (Conidi, Rodriguez‐Lopez, Garcia‐Castello, & Cassano, 2015; Verma & Sarkar 2015). Compared with the flux changes on the same MWCO of M1, M2, and M3, membrane material also affected on the flux significantly. The flux of M2, made from PVDF, decreased significantly, from 83.51 to 19.57 L/(m^2^·hr).

Effects of TMP and CFR on flux are shown in Figure 2b–d. The rapid decline of flux at the initial stage had a direct relationship with TMP. Figure 2b shows that when the CFR and TMP were 15 L/hr and 0.25 MPa, the flux dropped from 15.07 to 8.53 L/(m^2^·hr) in the first 5 min. Flux decreased faster in the process at a higher TMP. As reported by Qaid, Zait, and Taky (2016) and Benítez, Acero, Leal, and González (2009), this phenomenon was due to the cake layer which has not yet formed at the initial stage, in which the membrane permeability resistance was the main resistance. In Figure 2c, when the TMP increased from 0.25 to 0.75 MPa, the flux increased from 22.15 to 43.45 L/(m^2^·hr).

Cross flow rate can also play an important role in flux, especially at a lower TMP. The flux can be improved when the CFR increased, caused by the increased shear force near membrane surface. This can slow down the formation of cake layer. Figure 2b,d show that the flux increased from 7.65 to 35.81 L/(m^2^·hr) after 30 min when the CFR rose from 15 to 45 L/hr. It demonstrates that the improvement effects of increased CFR on flux decreased as the TMP increased. This may be due to the change of the configuration of higher molecular weight components such as proteins or polysaccharides at a high pressure condition (Mondal, Biswas, & De, 2016).

Accordingly, various conditions can affect the permeate flux because of the changes of fouling degree on membranes. Different fouling degrees make the components in the juices formed on the membrane surface differences, which can also affect the characteristics of apple juices.

### 3.2 Effects of various conditions on quality of clarified apple juice

Table 2 shows the properties of apple juice treated by different UF membranes. With the same MWCO, ultrafiltrated juice obtained by M2 has the highest phenolic content and lowest proteins content. While ultrafiltrated juice obtained by M3 has the highest color, this phenomenon can be attributed to the different membrane materials. It indicates that with the increase of MWCO, total phenols, total proteins, total sugars, and total soluble solids in clarified apple juice increased with the same membrane types. Total sugars in the filtrate treated by M3 were about 2.78 times of that by M5. Because the differences of a cake layer formed by some macromolecules such as proteins, polysaccharides, and aggregates on membrane surface. This cake layer will intercept some small molecular chemicals, such as phenols and monosaccharide. As Huang et al. (2013) reported that with the increase of MWCO, when solutes with larger size that have been trapped on membrane surface to form a cake layer, the layer composed of polymer solutes has more holes and higher permeability. Concentration of protein can also affect the shelf life of fruit juice. Low protein content of apple juice treated with M4 was beneficial for storage, and the other components were higher. Meanwhile, the high permeate flux made the process more effective. Therefore, M4 was selected as the best membrane for clarification.

**TABLE 2:** Physicochemical properties and quality of apple juice clarified by different UF membranes

No.	Color (A_420_)	Clarity (%*T*)	Phenolic (μg/ml)	Protein (μg/ml)	Sugar (mg/ml)	TSS (°Brix)	pH
M1	0.12 ± 0.03^a^	96.84 ± 0.16^b^	80.2 ± 1.21^c^	60.81 ± 4.20^d^	59.46 ± 0.30^b^	9.10 ± 0.14^b^	3.8 ± 0.1^a^
M2	0.18 ± 0.01^b^	96.71 ± 0.47^a^	90.4 ± 1.10^c^	52.41 ± 3.10^c^	61.78 ± 0.22^c^	10.80 ± 0.28^c^	3.9 ± 0.1^a^
M3	0.28 ± 0.02^a^	96.38 ± 0.32^c^	83.5 ± 3.07^a^	54.15 ± 2.01^c^	70.90 ± 0.29^d^	11.05 ± 0.21^d^	3.9 ± 0.2^a^
M4	0.08 ± 0.00^c^	97.38 ± 0.47^c^	43.4 ± 2.19^b^	24.13 ± 1.04^b^	47.89 ± 0.11^b^	8.60 ± 0.14^b^	4.0 ± 0.2^b^
M5	0.06 ± 0.00^a^	98.52 ± 0.16^b^	33.3 ± 2.13^a^	13.29 ± 0.29^a^	25.45 ± 0.13^a^	7.50 ± 0.14^a^	4.1 ± 0.3^b^
Feed	0.71 ± 0.04^b^	88.41 ± 0.14^a^	190.2 ± 5.40^d^	0.15 ± 0.01^c^	104.81 ± 0.45^d^	12.60 ± 0.28^b^	3.8 ± 0.2^c^

Note

Values followed by different superscripts within each column are significantly different (*p* < .05).

As shown in Table 3, profiles of apple juice among nine groups at different conditions were almost the same. When the flow rate and pressure increasing, total phenols, total sugars, and total soluble solids all increased except the total proteins. Clarification degree decreased as the pressure increasing. Consequently, the optimal conditions for apple juice clarification should be at 30 L/hr and 0.75 MPa.

**TABLE 3:** Physicochemical properties and quality of apple juice clarified at different UF processes

No.	CFR (L/hr)	TMP (MPa)	Color (A_420_)	Clarity (%*T*)	Phenolic (μg/ml)	Protein (μg/ml)	Sugar (mg/ml)	TSS (°Brix)	pH
1	15	0.25	0.09 ± 0.01^a^	98.06 ± 0.16^c^	43.50 ± 2.10^a^	20.30 ± 0.08^b^	46.87 ± 0.15^a^	8.40 ± 0.28^a^	4.3 ± 0.1^c^
2	15	0.50	0.08 ± 0.00^a^	97.38 ± 0.47^b^	45.11 ± 1.90^a^	24.20 ± 0.15^b^	48.89 ± 0.11^b^	8.60 ± 0.14^a^	4.0 ± 0.2^ab^
3	15	0.75	0.13 ± 0.01^b^	97.38 ± 0.47^b^	50.32 ± 2.11^ab^	14.31 ± 0.19^a^	49.66 ± 0.27^bc^	9.50 ± 0.14^b^	4.1 ± 0.1^b^
4	30	0.25	0.12 ± 0.00^b^	97.61 ± 0.48^b^	54.44 ± 2.21^ab^	32.17 ± 1.01^c^	48.94 ± 0.13^b^	8.85 ± 0.21^a^	4.2 ± 0.1^b^
5	30	0.50	0.12 ± 0.00^b^	97.61 ± 0.16^b^	60.71 ± 1.10^b^	23.28 ± 0.62^b^	49.42 ± 0.12^bc^	9.35 ± 0.07^b^	4.2 ± 0.2^b^
6	30	0.75	0.15 ± 0.00^c^	96.94 ± 0.16^a^	61.30 ± 2.02^b^	13.16 ± 1.23^a^	50.70 ± 0.26^bc^	9.55 ± 0.07^b^	4.2 ± 0.1^b^
7	45	0.25	0.23 ± 0.00^d^	97.05 ± 0.31^ab^	54.22 ± 1.20^ab^	22.12 ± 1.20^b^	59.55 ± 0.24^d^	9.40 ± 0.14^b^	4.0 ± 0.2^ab^
8	45	0.50	0.16 ± 0.00^c^	97.05 ± 0.31^ab^	62.00 ± 1.10^b^	13.35 ± 0.19^a^	52.43 ± 0.31^c^	10.75 ± 0.07^c^	3.9 ± 0.1^a^
9	45	0.75	0.19 ± 0.00^cd^	96.49 ± 0.16^a^	73.21 ± 3.30^c^	14.12 ± 1.02^a^	52.65 ± 0.25^c^	11.05 ± 0.07^d^	3.8 ± 0.2^a^
Feed	15	0.5	0.71 ± 0.04^c^	88.41 ± 0.14^c^	190.12 ± 5.04^d^	154.02 ± 6.01^a^	104.81 ± 0.45^a^	12.60 ± 0.28^b^	3.8 ± 0.2^b^

Note

Values followed by different superscripts within each column are significantly different (*p* < .05).

### 3.3 Effects of UF on polyphenol profiles of apple juice

**FIGURE 3:**
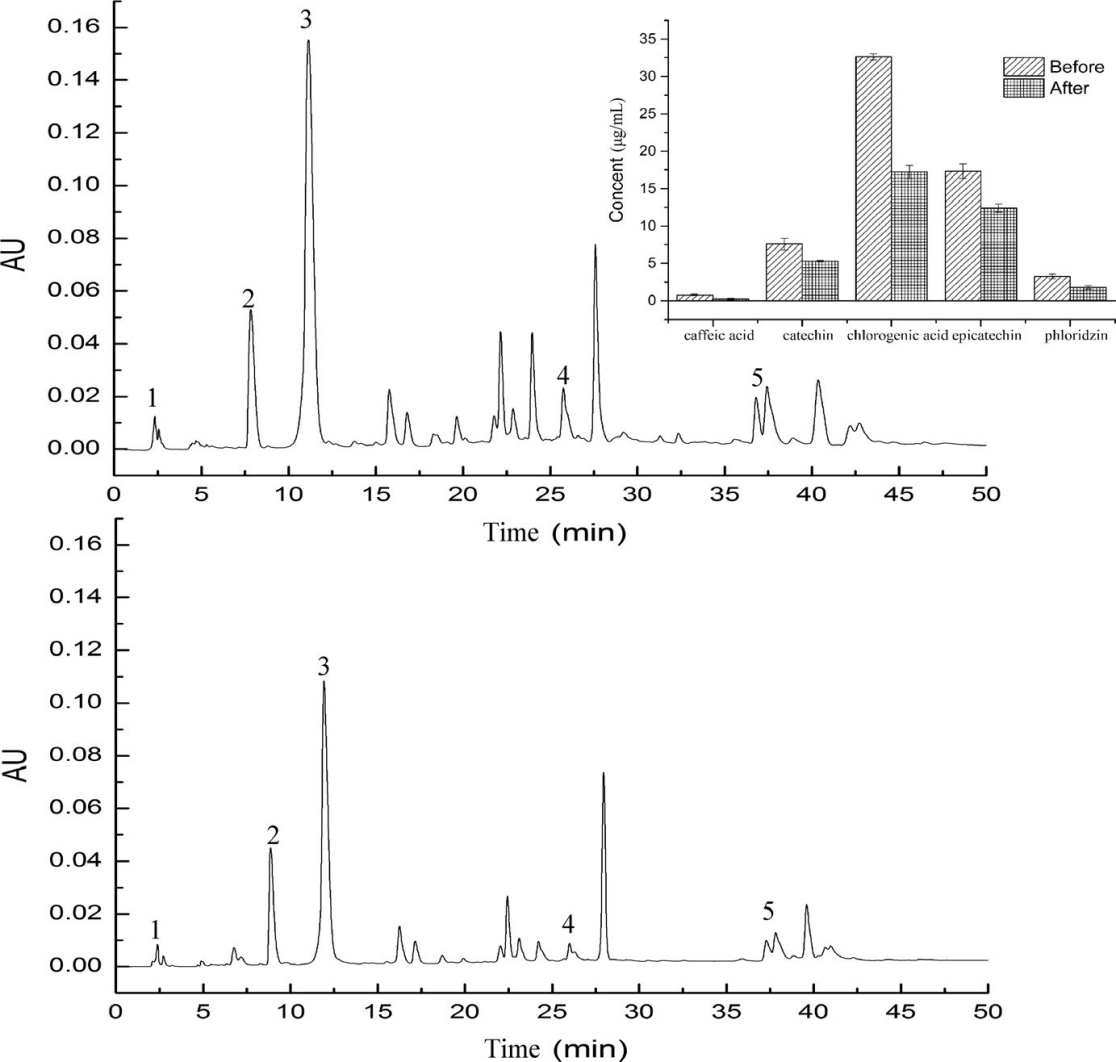
Changes in polyphenols before (a) and after (b) ultrafiltration. 1. caffeic acid; 2. catechin; 3. chlorogenic acid; 4. epicatechin; 5. phloridzin

Polyphenols in apple juice might be combined with proteins, or co‐colored with other compounds in the system, or oxidative condensation of polyphenols themselves. Other components in apple juice may also be directly or indirectly affected with polyphenols. As shown in Figure 3, after UF with M4 at 30 L/hr and 0.75 MPa, some polyphenols in the permeate decreased significantly, in which chlorogenic acid decreased about 47.16%, from 32.63 to 17.24 μg/ml, epicatechins about 28.56%, from 17.33 to 12.38 μg/ml, phloridzin about 44.92%, from 3.25 to 1.79 μg/ml, catechin about 30.47%, from 7.58 to 5.27 μg/ml and caffeic acid about 66.6%, from 0.75 to 0.25 μg/ml. These decreases might be caused by the interactions of polyphenols with membrane materials or the cake layer during ultrafiltration (Baklouti, Ellouze‐Ghorbel, Mokni, & Chaabouni, 2012).

### 3.4 Properties change of clarified juice during storage

Table 4 shows the changes of quality properties of clarified apple juice in 4 weeks storage. It indicates that the properties changed slightly. Clarity, total sugars, and total phenols reduced by 2.5%, 6.4%, and 16.6%, respectively, while TSS increased by 3.1%. Concentration of phenolics decreased gradually, this degradation of polyphenols was accordance with the reported study (Knebel, Braun, & Dietrich, 2018). Consequently, ultrafiltrated juice can be preserved at 4°C without significant quality deterioration for 4 weeks.

**TABLE 4:** Physicochemical properties and quality of clarified apple juice during storages

Weeks	Color (A_420_)	Clarity (%*T*)	Phenolic (μg/ml)	Protein (μg/ml)	Sugar (mg/ml)	TSS (°Brix)	pH
0	0.15 ± 0.00^a^	96.94 ± 0.16^a^	64.12 ± 0.91^b^	13.20 ± 0.08^a^	50.70 ± 0.26^a^	9.55 ± 0.07^a^	4.2 ± 0.1^a^
1	0.16 ± 0.01^c^	96.69 ± 0.14^b^	63.12 ± 0.81^c^	14.3 ± 0.00^b^	50.65 ± 0.16^c^	9.75 ± 0.20^b^	3.9 ± 0.2^a^
2	0.17 ± 0.02^a^	95.56 ± 0.26^c^	54.31 ± 2.02^c^	14.5 ± 0.81^c^	49.26 ± 0.34^b^	9.80 ± 0.10^b^	3.8 ± 0.1^b^
3	0.17 ± 0.01^c^	94.61 ± 0.13^d^	53.21 ± 0.90^a^	19.5 ± 1.20^d^	49.12 ± 0.28^b^	9.85 ± 0.10^c^	3.9 ± 0.1^c^
4	0.17 ± 0.00^c^	94.52 ± 0.18^a^	52.10 ± 0.01^b^	20.2 ± 0.11^c^	47.48 ± 0.38^c^	9.85 ± 0.20^c^	3.8 ± 0.2^c^

Note

Values followed by different superscripts within each column are significantly different (*p* < .05).

## 4 CONCLUSIONS

Clarified apple juice is popular for consumers because of its unique light transmittance, flavor, and taste. An optimal membrane and operated conditions carried out could promote the quality of clarified juice. PES‐10 kDa membrane, CFR 30 L/hr, and TMP 0.75 MPa were found to be the most suitable conditions for clarification of apple juice. The clarified apple juice with a color 0.15 A_420_, clarity 96.94%*T*, TSS 9.55 °Brix, pH value 4.2, and total phenols, total proteins, and total sugars were 64.12 and 13.20 μg/ml and 50.70 mg/ml, respectively. Ultrafiltrated juice can be preserved at 4°C without significant quality deterioration for 4 weeks. However, there are still some components loss during the clarification process. It is necessary to find a way to improve the membrane technology for juice treatment.

## CONFLICT OF INTEREST

The authors declared that we had no any conflict of interest.

## ETHICAL APPROVAL

The study did not include any animal or human tests.
